# Supplementary material for: Efficacy of intranasal adenovirus vector vaccine is attenuated by type I IFN-induced NK cell activation
Source: Mol Ther Adv. 2026 Apr 24;34(2):201742. doi: 10.1016/j.omta.2026.201742 (PMC13186022; doi:10.1016/j.omta.2026.201742)
Supplement: Document S1. Figures S1–S4 [file mmc1.pdf]

OMTA, Volume *34*

Supplemental information

Efficacy of intranasal adenovirus vector vaccine is attenuated by type I IFN-induced NK cell activation

Hayato Nakatani, Masashi Tachibana, Rika Onishi, Takato Nakagaki, Ken J. Ishii, Kahori Shimizu, Fuminori Sakurai, and Hiroyuki Mizuguchi

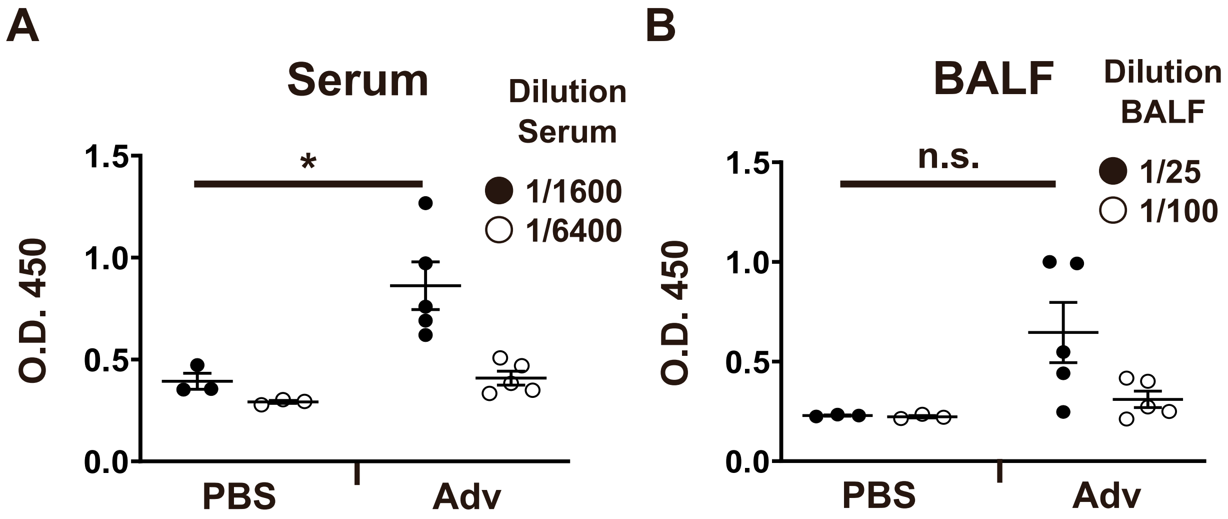

Figure S1. The induction of anti-Ad IgG in serum and BALF following *i.n.* Adv vaccination. (A, B) WT mice were administered Ad-LacZ *i.n*. at a dose of 1.0 × 10^{10} VP/mouse on days 0 and 14. Titers of anti-Ad IgG in (A) serum and (B) BALF on day 21 were measured by ELISA using 96-well plates coated with a replication-incompetent Ad5 vector encoding no transgene (5 × 10^{6} VP/well). Data are pooled (A, B) from one independent experiment and are shown as the mean ± S.E.M. (A, B) PBS (n = 3), Adv (n = 5). *$p < 0.05$.

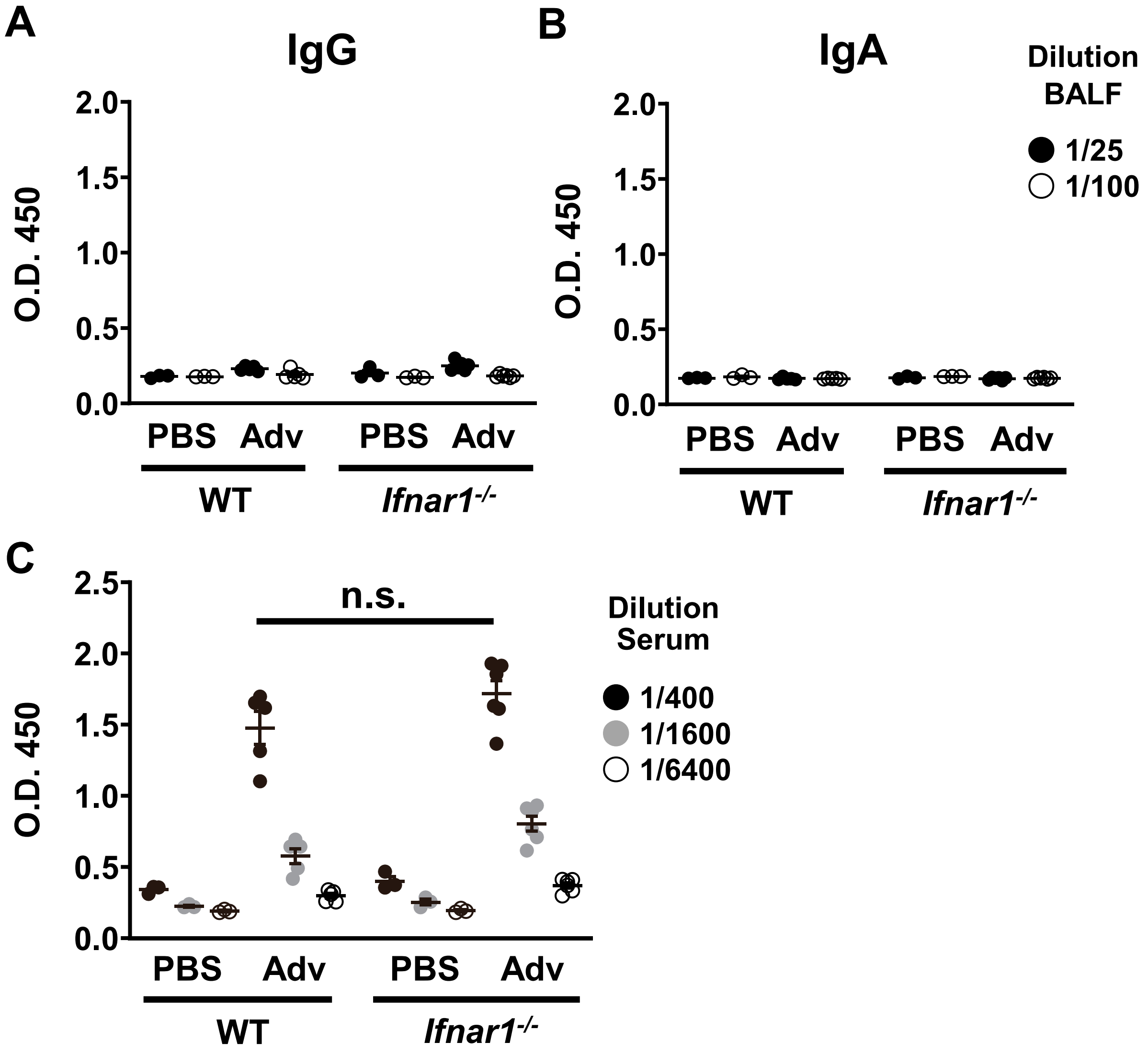

Figure S2. Type I IFN signaling does not contribute to β-gal–specific antibody production at respiratory mucosal surfaces following *i.m.* Adv vaccination. (A-C) WT and *Ifnar1*$^{-/-}$ mice were administered Ad-LacZ *i.m.* at a dose of 1.0 × 10^{10} VP/mouse on days 0 and 14. Titers of anti-β-gal (A) IgG and (B) IgA in BALF and of (C) anti-β-gal IgG in serum were measured on day 21. Data are pooled (A-C) from two independent experiments and are shown as the mean ± S.E.M. (A-C) PBS (n = 3), WT + Ad (n = 5), *Ifnar1*$^{-/-}$ + Ad (n = 6).

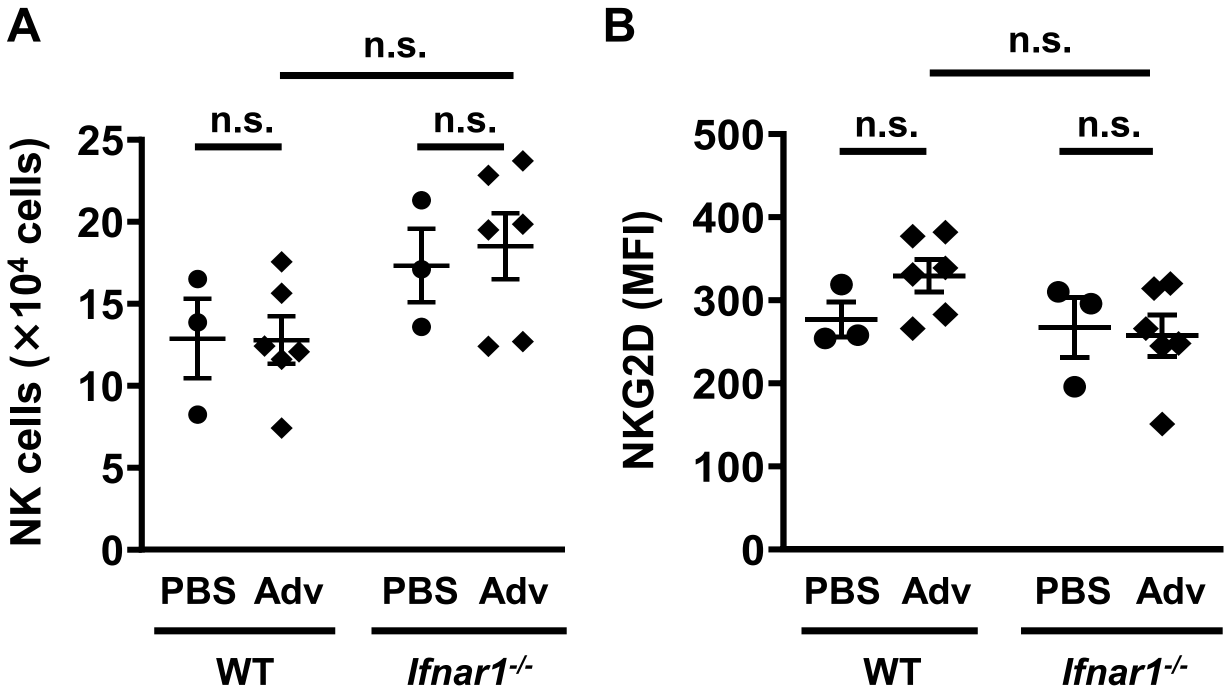

Figure S3. NK cells were not activated in the spleen after *i.n.* Adv vaccination. (A, B) WT and *Ifnar1*$^{-/-}$ mice were *i.n.* administered Ad-LacZ at a dose of 1.0×10^{10} VP/mouse on day 0. In the spleen after 3 days of *i.n.* administration, (A) NK cell (NK1.1^{+}CD3^{-}) number, and (B) the expression of NKG2D, assessed by mean fluorescence intensity (MFI) as a marker of NK cell activation, were measured by flow cytometry. Data are pooled (A, B) from two independent experiments and are shown as the mean ± S.E.M. (A, B) PBS (n = 3), Ad (n = 6). *$p < 0.05$.

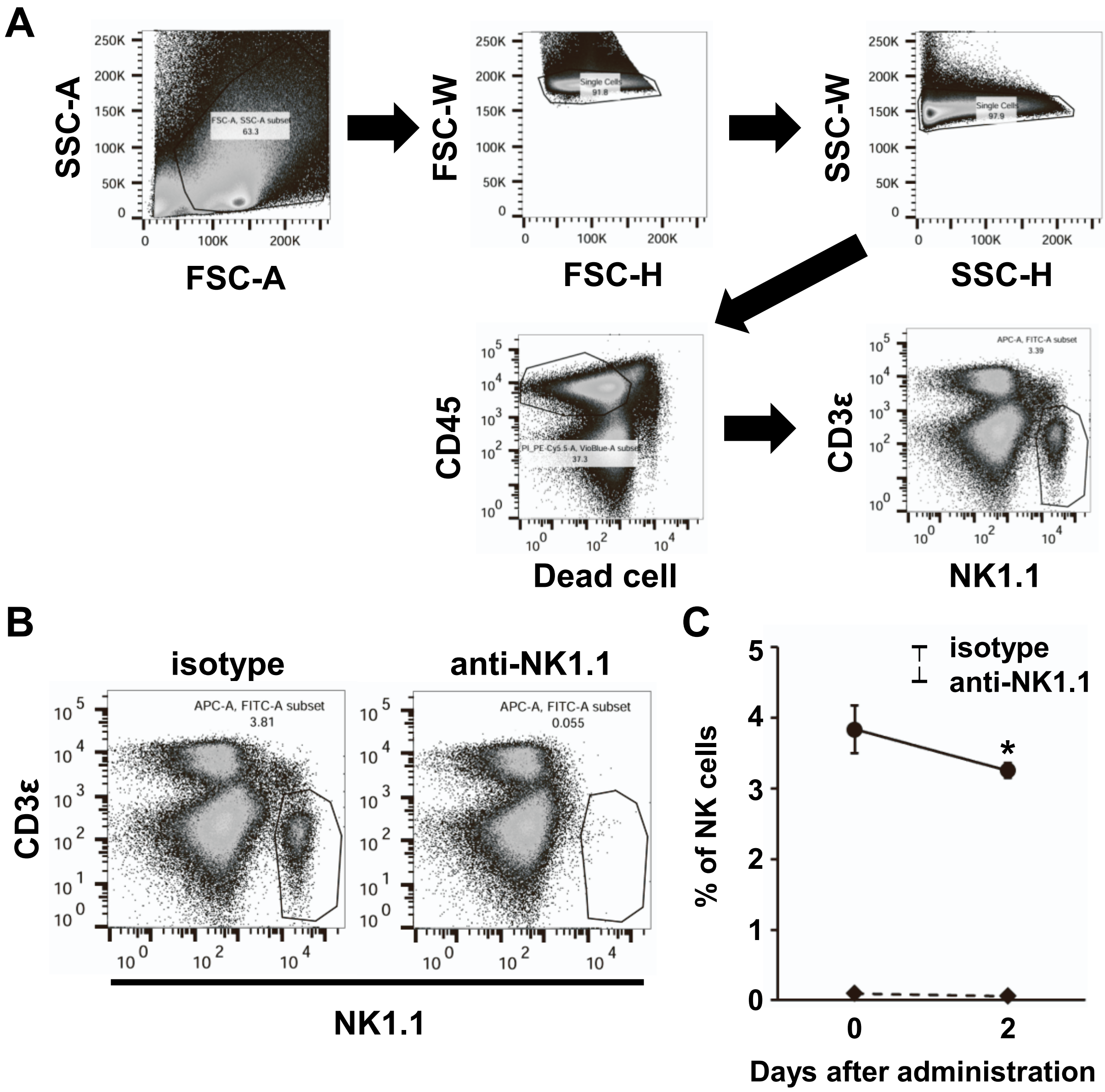

Figure S4. Flow cytometry gating strategy and NK cell depletion in the lung. (A) The flow cytometry gating strategy is illustrated. (B) NK cell populations in the lungs of WT mice treated with either IgG2a isotype control or anti-NK1.1 were compared. (C) WT mice were treated with isotype control or anti-NK1.1 on day -1. Lungs were excised on days 0 and 2, and the percentage of NK cells among $CD45^+$ cells was measured by flow cytometry. Data are pooled (C) from two independent experiments and are shown as the mean ± S.E.M. (C) isotype ($n = 3$-4), anti-NK1.1 ($n = 4$). *$p < 0.05$.
