# Supplementary material for: Efficacy of intranasal adenovirus vector vaccine is attenuated by type I IFN-induced NK cell activation
Source: Mol Ther Adv. 2026 Apr 24;34(2):201742. doi: 10.1016/j.omta.2026.201742 (PMC13186022; doi:10.1016/j.omta.2026.201742)
Supplement: Document S2. Article plus supplemental information [file mmc2.pdf]

Molecular Therapy
Advances
Original Article

Efficacy of intranasal adenovirus vector vaccine is attenuated by type I IFN-induced NK cell activation

Hayato Nakatani,[1] Masashi Tachibana,[1,2] Rika Onishi,[1] Takato Nakagaki,[3] Ken J. Ishii,[4,5,6] Kahori Shimizu,[1,3] Fuminori Sakurai,[1,7,8] and Hiroyuki Mizuguchi[1,3,9,10,11]

[1]Laboratory of Biochemistry and Molecular Biology, Graduate School of Pharmaceutical Sciences, The University of Osaka, Osaka 565-0871, Japan; [2]Laboratory for Context-dependent Cell Immunology, Department of Biomedical Sciences, College of Life Sciences, Ritsumeikan University, Shiga 525-8577, Japan; [3]Laboratory of Biochemistry and Molecular Biology, School of Pharmaceutical Sciences, The University of Osaka, Osaka 565-0871, Japan; [4]Division of Vaccine Science, Department of Microbiology and Immunology, The Institute of Medical Science, The University of Tokyo, Tokyo 108-8639, Japan; [5]International Vaccine Design Center, The Institute of Medical Science, The University of Tokyo, Tokyo 108-8639, Japan; [6]Immunology Frontier Research Center, The University of Osaka, Osaka 565-0871, Japan; [7]Laboratory of Biopharmaceutics, Faculty of Pharmacy, Kindai University, Osaka 577-0813, Japan; [8]Pharmaceutical Research and Technology Institute, Kindai University, Osaka 577-0813, Japan; [9]The Center for Advanced Medical Engineering and Informatics, The University of Osaka, Osaka 565-0871, Japan; [10]Integrated Frontier Research for Medical Science Division, Institute for Open and Transdisciplinary Research Initiatives (OTRI), The University of Osaka, Osaka 565-0871, Japan; [11]Center for Infectious Disease Education and Research (CiDER), The University of Osaka, Osaka 565-0871, Japan

Adenovirus vector (Adv)-based intranasal (i.n.) vaccines induce mucosal immunity and are thus a promising strategy against various infectious diseases. To improve the safety and efficacy of Adv vaccines, it is essential to clarify the mechanisms underlying the induction of immune responses. We focused on type I interferons (IFNs), which play a crucial role in the antiviral response. To elucidate the mechanisms behind i.n. Adv vaccination, we intranasally administered antigen (Ag)-expressing Adv to type I IFN receptor-deficient (*Ifnar1*$^{-/-}$) mice. Our findings indicated that type I IFN signaling specifically suppresses Ag-specific antibody production at the airway mucosal surface. Depletion of NK cells revealed that low Ag expression results from enhanced NK cell activity, which eliminates Adv-infected cells, thereby reducing Ag-specific antibody production at the airway mucosal surface. Together, these results suggest that type I IFN signaling decreases Ag expression by promoting the elimination of Adv-infected cells via NK cell activation, which attenuates Ag-specific antibody production during i.n. Adv vaccination. Our findings contribute to the development of more potent and optimized Adv vaccines.

INTRODUCTION

Numerous emerging and reemerging infectious diseases, such as coronavirus disease 2019 (COVID-19), influenza, and measles, have emerged worldwide in the last decade. Because respiratory viruses infect the body through mucosal surfaces, vaccines must strongly induce both a mucosal immune response to protect against pathogen entry and a systemic immune response to eliminate invading pathogens.[1,2] Intramuscular (i.m.) administration, commonly used in vaccinations, can induce immune responses in systemic compartments, but not in the mucosa. In contrast, intranasal (i.n.) administration is expected to induce antigen (Ag)-specific immune responses in both systemic compartments and at the airway mucosal surface.[3]

FluMist (AstraZeneca), an i.n. live-attenuated vaccine, has been reported to be highly effective in preventing seasonal influenza infections in children and young adults except for older adults.[4] However, live-attenuated vaccines raise safety concerns due to the potential for genetic reversion to pathogenic viruses. To address this issue, we focused on replication-incompetent recombinant adenovirus vector (Adv) vaccines, which serve as safe gene delivery vectors due to their low genotoxicity, as they do not integrate genetic material into chromosomal DNA. Adv vaccines can induce strong immunity by promoting high expression of the Adv-expressing Ag gene and can also induce Ag-specific mucosal immune responses through i.n. administration. In addition, unlike live-attenuated vaccines, Adv vaccines are well suited for rapid deployment against various infectious diseases since the inserted gene can be easily replaced with different Ags. Consistent with these properties, some of the i.n. Adv vaccines have already been approved. CanSino Biologics and Bharat Biotech have developed inhaled COVID-19 vaccines, Convidecia Air and iNCOVACC, respectively.[5,6] These approved vaccines underscore the translational potential of Adv as an i.n. vaccine platform. These advantages position Adv as a candidate for a potent and safe i.n. vaccine modality to combat pandemics.

Received 18 September 2025; accepted 21 April 2026;
https://doi.org/10.1016/j.omta.2026.201742.

Correspondence: Hiroyuki Mizuguchi, Laboratory of Biochemistry and Molecular Biology, Graduate School of Pharmaceutical Sciences, The University of Osaka. 1-6 Yamadaoka, Suita, Osaka 565-0871, Japan.
E-mail: mizuguch@phs.osaka-u.ac.jp

© 2026 The Author(s).
Published by Elsevier Inc. on behalf of The American Society of Gene and Cell Therapy.
This is an open access article under the CC BY license (http://creativecommons.org/licenses/by/4.0/).

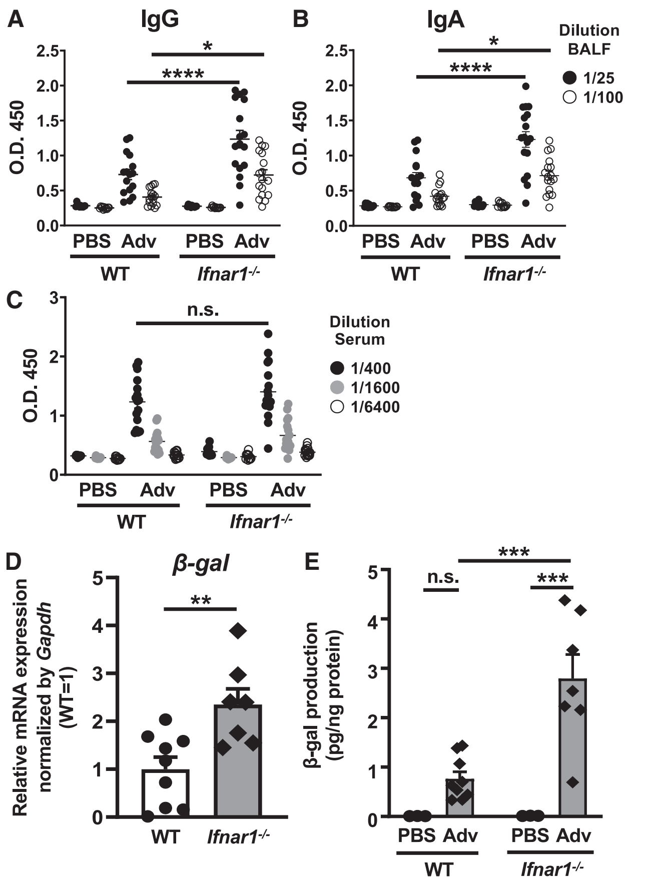

Figure 1. Suppression of β-gal-specific antibody production in the BALF via type I IFN signaling following i.n. Adv vaccination

(A–C) WT and *Ifnar1*$^{-/-}$ mice were administered Ad-LacZ intranasally at a dose of 1.0×10^{10} VP/mouse on days 0 and 14. Titers of anti-β-gal (A) IgG and (B) IgA in BALF and of (C) anti-β-gal IgG in the serum on day 21 were measured. (D and E) β-Gal expression in the lung was measured at the mRNA and protein levels. (D) At 48 h after i.n. Ad-LacZ vaccination, total RNA was extracted from the lungs, and mRNA expression of β-gal was measured by RT-qPCR and normalized to *Gapdh* expression. Graphs represent the relative mRNA expression of each gene normalized to its expression in WT mice. (E) β-Gal activity levels in the lung were determined 48 h after administration. Data are pooled from (A–C) four and (D and E) two independent experiments and are shown as the mean ± SEM. (A–C) PBS (n = 11), WT + Ad (n = 16), *Ifnar1*$^{-/-}$ + Ad (n = 17); (D) WT (n = 9), *Ifnar1*$^{-/-}$ (n = 7); and (E) PBS (n = 3), WT + Ad (n = 9), *Ifnar1*$^{-/-}$ + Ad (n = 7). $^{*}p < 0.05$, $^{**}p < 0.01$, $^{***}p < 0.001$, $^{****}p < 0.0001$.

The induction of pathogen-specific acquired immunity requires not only Adv-mediated Ag expression but also the activation of innate immunity by adjuvants.[7–9] In Adv vaccines, viral components such as capsid proteins, genomic DNA, and non-coding RNA are recognized by innate immune receptors, triggering innate immune responses.[10–15] This results in the production of type I interferons (IFNs) and inflammatory cytokines. Indeed, Adv infection has been shown to induce the production of type I IFNs, which in turn promote the expression of various cytokines and interferon-stimulated genes (ISGs), leading to antiviral effects such as the suppression of viral replication, enhancement of dendritic cell function, and activation of natural killer (NK) cells.[15–18] Thus, Adv represents a promising modality that can deliver Ags while providing potent adjuvant activity, capable of generating a strong vaccine effect. However, the innate immune signals critical for inducing Ag-specific acquired immunity following i.n. administration of Adv remain unclear. To establish an i.n. vaccine platform based on Adv, it is essential to optimize Ag expression and immunogenic properties while elucidating the mechanisms of innate immune activation and Ag-specific acquired immune responses following i.n. administration.

In this study, we focused on type I IFNs, which are crucial for antiviral responses, to elucidate the mechanisms underlying the induction of vaccine effects following i.n. administration of Adv vaccines. We found that type I IFN signaling attenuates Ag-specific antibody production through the activation of NK cells. Our findings support the development of more effective Adv vaccines capable of establishing protective immunity in both systemic compartments and the airway mucosal surface.

RESULTS

Type I IFN signaling attenuates Ag-specific antibody production in bronchoalveolar lavage fluid following i.n. adv vaccination

To determine whether type I IFN signaling is involved in the Ag-specific antibody production, we measured β-galactosidase (β-gal)-specific antibody titers in bronchoalveolar lavage fluid (BALF) and serum after i.n. administration of the adenovirus type 5 vector expressing β-gal (Ad-LacZ). The results show that IgG and IgA titers in BALF were significantly higher in type I IFN receptor-deficient (*Ifnar1*$^{-/-}$) mice than in wild-type (WT) mice, while IgG titers in serum were similar in both groups (Figures 1A–1C). Anti-Ad IgG titers in BALF and serum after i.n. administration of the Ad-LacZ were induced similar to the anti-β-gal IgG titers (Figures S1A and S1B). These results suggest that type I IFN signaling specifically suppresses β-gal-specific antibody production at the airway mucosal surface.

Type I IFNs are known to promote the elimination of Adv-infected cells through the activation of innate and adaptive immunity,[19–21] thereby decreasing Adv-encoded Ag gene expression. We hypothesized that the increased β-gal-specific antibody production observed in *Ifnar1*$^{-/-}$ mice may result from elevated β-gal gene expression. We analyzed β-gal gene expression levels in the lungs after i.n. administration of Ad-LacZ. The results indicate that both mRNA and protein levels of β-gal were significantly higher in *Ifnar1*$^{-/-}$ mice than in WT mice (Figures 1D and 1E). These findings suggest that type I IFN signaling decreases Ag gene expression in the lungs, resulting in lower Ag-specific antibody production in BALF following i.n. Adv vaccination.

We also measured β-gal-specific antibody titers in BALF and serum after i.m. administration of Ad-LacZ. The results show that IgG and IgA titers in BALF of both WT and *Ifnar1*$^{-/-}$ mice were not induced, while IgG titers in serum remained at similar levels in both groups (Figures S2A–S2C). Thus, these findings suggest that Ag-specific

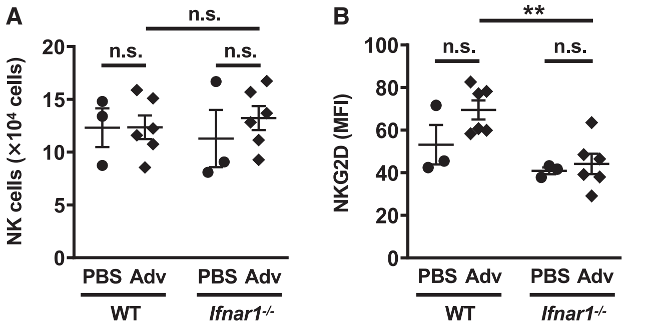

Figure 2. Type I IFN signaling enhances NK cell activity in the lung after i.n. Adv vaccination

(A and B) WT and *Ifnar1*$^{-/-}$ mice were administered Ad-LacZ intranasally at a dose of 1.0×10^{10} VP/mouse on day 0. In the lung, 3 days after i.n. administration, (A) the number of NK cells (NK1.1$^+$CD3ε$^-$) and (B) the expression of NKG2D, assessed by mean fluorescence intensity (MFI) as a marker for NK cell activation, were measured by flow cytometry. Data are pooled (A and B) from two independent experiments and are shown as the mean ± SEM. (A and B) PBS ($n = 3$), Ad ($n = 6$). $^{**}p < 0.01$.

antibody production after i.m. Adv vaccination is independent of type I IFN signaling.

Type I IFN signaling induces NK cell activation in the lung following i.n. Adv vaccination

We hypothesized that Adv-infected cells might be eliminated by NK cells activated through type I IFN signaling, leading to a decrease in Ag gene expression. To test this, we examined the number and activation of NK cells in the lungs of WT and *Ifnar1*$^{-/-}$ mice following i.n. administration of Ad-LacZ. We found that the expression of NKG2D, an activation marker, was significantly lower in *Ifnar1*$^{-/-}$ mice compared to WT mice, while the number of NK cells was similar between both groups (Figures 2A and 2B).

Previous study has indicated that C-X-C motif chemokine receptor 3 (CXCR3) on NK cells is involved in their recruitment.[22] In addition, since activated NK cells express various cytotoxic factors such as granzyme B, perforin, and proinflammatory cytokines including IFN-γ, we hypothesized that these factors may be induced in a type I IFN-dependent manner.[23] We measured the expression of CXCR3 ligands and NK cell-related genes in the lungs after i.n. administration of Ad-LacZ. The expression of CXCR3 ligands (*Cxcl9*, *Cxcl10*, and *Cxcl11*) was upregulated in the lungs of WT mice, but not of *Ifnar1*$^{-/-}$ mice (Figure 3A). Conversely, *Gzmb* and *Ifng* expression was not upregulated in the lungs of *Ifnar1*$^{-/-}$ mice following Ad-LacZ administration, while *Prf1* expression was not upregulated in either WT or *Ifnar1*$^{-/-}$ mice (Figure 3B). These results suggest that the decrease in Ag gene expression due to type I IFN signaling following i.n. Adv vaccination is attributable to the promotion of Adv-infected cell elimination through type I IFN-induced NK cell activation rather than the recruitment of NK cells into the lungs.

To determine whether NK cell activation via type I IFN signaling occurs in the spleen, we examined the number and activation of NK cells in the spleens of WT and *Ifnar1*$^{-/-}$ mice after i.n. administration of Ad-LacZ. We found that both the number of NK cells and the expression of NKG2D were comparable between WT and *Ifnar1*$^{-/-}$ mice (Figures S3A and S3B). These findings suggest that NK cell activation following i.n. Adv vaccination may be restricted to the lungs.

Elimination of Adv-infected cells by NK cells causes low Ag-specific antibody production in BALF following i.n. adv vaccination

To determine whether NK cells, which may eliminate Adv-infected cells, are essential for suppressing Ag-specific antibody production following i.n. administration of Adv, we administered anti-NK1.1 antibody intraperitoneally to evaluate the effect of NK cell depletion. The results indicate that IgG and IgA titers in the BALF were significantly higher in NK cell-depleted mice compared to control mice, while IgG titers in serum were similar in both groups (Figures 4A–4C).

To assess whether β-gal-specific antibody production correlates with Ag gene expression, we analyzed β-gal gene expression levels in the lungs after i.n. administration of Ad-LacZ. The results demonstrate that both mRNA and protein levels were significantly higher in NK cell-depleted mice than in control mice (Figures 4D and 4E), suggesting that NK cells suppress β-gal gene expression in the lungs. These findings imply that the early elimination of Adv-infected cells by NK cells reduces Ag gene expression in the lungs, thereby suppressing Ag-specific antibody production in the BALF following i.n. Adv vaccination.

DISCUSSION

i.n. Adv vaccines are anticipated to provide an effective defense against infectious diseases due to their capacity to induce both systemic and mucosal immunity. However, the mechanisms underlying the immune response induced by *i.n.* Adv vaccination remain unclear. Uncovering these mechanisms is crucial for enhancing the safety and efficacy of i.n. Adv vaccines. In this study, we demonstrated that type I IFN signaling reduces Ag expression by eliminating Adv-infected cells through NK cell activation, which in turn attenuates Ag-specific antibody production during i.n. Adv vaccination.

The 30-μL i.n. dose used in this study is generally considered a relatively large-volume i.n. administration in mice and is believed to result in Ag delivery not only to the upper respiratory tract but also to the lower respiratory tract (LRT). Previous studies have shown that vaccine delivery to the LRT by large-volume i.n. administration induces stronger immunogenicity and more sustained humoral immune responses compared to small-volume i.n. doses restricted to the nasal cavity.[24,25] Thus, it is important to note that this model better represents vaccines targeting the lungs, rather than purely i.n. vaccines as might be assumed for humans.

We demonstrated that type I IFN signaling suppresses Ag-specific antibody production following i.n. Adv vaccination at airway

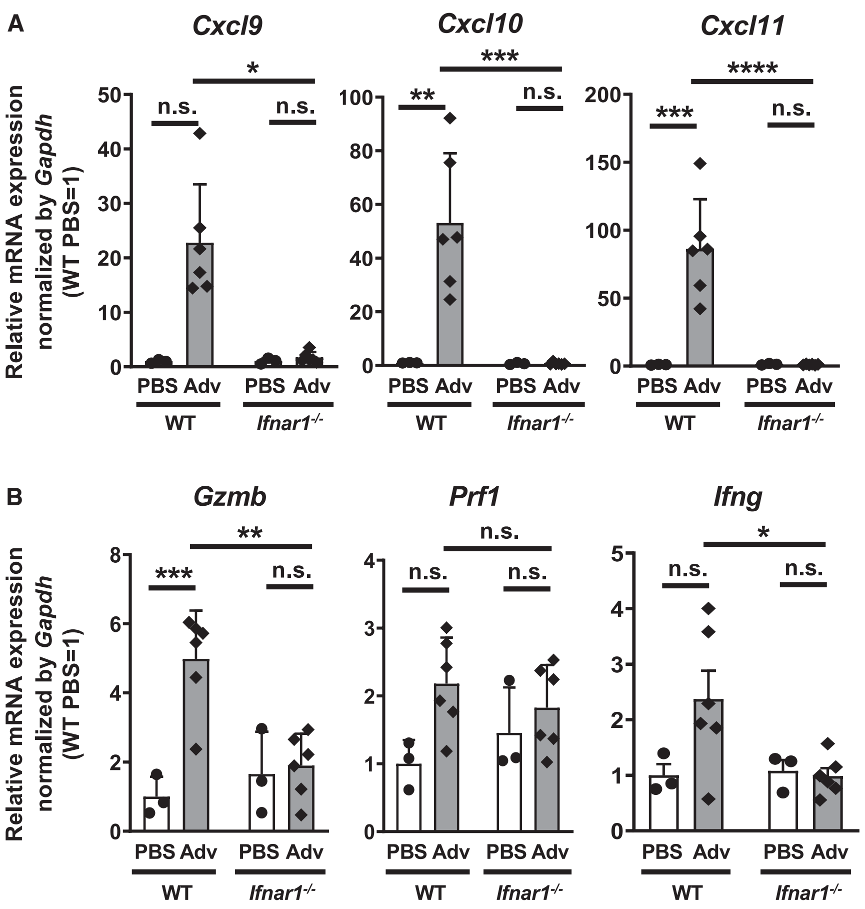

Figure 3. Inhibition of type I IFN signaling downregulates NK cell-related gene expression in the lung after i.n. Adv vaccination

(A and B) At 24 h after i.n. Ad-LacZ vaccination, total RNA was extracted from lungs, and mRNA expressions of (A) *Cxcl9*, *Cxcl10*, and *Cxcl11* and (B) *Gzmb*, *Pfl1*, and *Ifng* were measured by RT-qPCR and normalized by *Gapdh* expression. Graphs represent the relative mRNA expression of each gene normalized against its expression in WT mice treated with PBS. Data are pooled (A and B) from two independent experiments and are shown as the mean ± SEM. (A and B) PBS (n = 3), Ad (n = 6). *$p < 0.05$, **$p < 0.01$, ***$p < 0.001$, ****$p < 0.0001$.

mucosal surfaces, but not after i.m. Adv vaccination. Differences in the local immune environment, including the number and composition of resident immune cells, would result in difference in type I IFN dependency. The relative paucity of resident immune cells in muscle likely limits type I IFN-dependent Ab production following i.m. Adv administration.[26,27] On the other hand, abundant immune cells in lung can form transient inducible bronchus-associated lymphoid tissue after infection,[28–30] which supports local Ag presentation and adaptive immune responses. In such a local environment, type I IFN signaling may suppress Ag-specific antibody production. Previous studies have demonstrated that type I IFN signaling can antagonize immune responses induced by Adv, particularly those based on non-Ad5 serotypes.[20,21] In addition, our findings reveal a suppressive role of type I IFN signaling in the context of an Ad5-based i.n. vaccine, acting primarily at the level of local transgene expression in lung rather than systemic immune priming. These suggest that the impact of type I IFN signaling on Adv vaccine effects depends on the level of type I IFN and the tissue environment. High levels of type I IFNs induced by several non-Ad5 serotypes could suppress immune responses regardless of local tissue environments, while the lower levels induced by Ad5 may only affect vaccine effects in lung. In addition, we found that NK cells are activated by i.n. Adv vaccination in a type I IFN-dependent manner, which is accompanied by increased expression of granzyme B and IFN-γ, but not of perforin. While perforin mRNA is typically expressed in NK cells under steady-state conditions, its translation is initiated upon NK cell activation.[31] Therefore, measuring perforin mRNA expression alone may not adequately assess NK cell activation, and protein-level analysis may be necessary.

Although type I IFN signaling following i.n. Adv vaccination upregulated the expression of CXCR3 ligands, NK cell recruitment into the lung did not occur. This implies that CXCR3 is not involved in NK cell recruitment. Together, these findings suggest that lung tissue-resident NK cells are responsible for eliminating Adv-infected cells. Reports indicate that a higher proportion of NK cells among tissue lymphocytes is found in the lung compared to the lymph nodes, bone marrow, spleen, blood, and liver, with more than 70% of lung NK cells exhibiting a mature phenotype ($CD11b^{high}$ $CD27^{low}$).[32] This indicates that NK cells in the lung can rapidly eliminate Adv-infected cells. Thus, our observations suggest that type I IFN signaling following i.n. Adv vaccination suppresses Ag-specific antibody production by facilitating the elimination of Adv-infected cells through the activation of lung-resident NK cells. Since NK cell activation by i.n. Adv vaccination was not observed in the spleen, it appears that NK cell activation via type I IFN signaling occurs exclusively in the lungs. Consequently, i.n. Adv vaccination is unlikely to cause systemic adverse effects, indicating its safety. These observations are further supported by clinical studies of i.n. Adv-based COVID-19 vaccines, such as Convidecia Air and iNCOVACC, which have demonstrated acceptable safety profiles in humans.

In this study, we evaluated the effects of NK cells only in the early stages following i.n. Adv vaccination; therefore, further investigation is needed to elucidate the contributions of B cells and T cells involved in acquired immunity. In addition, alveolar macrophages may also reduce the local availability of Ag in the early stages by phagocytosis

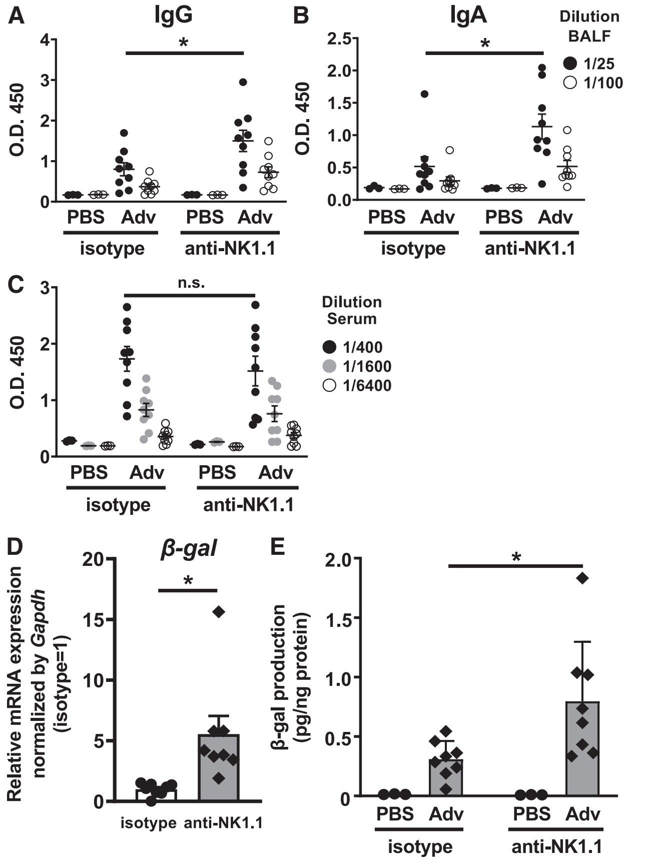

Figure 4. Suppression of β-gal-specific antibody production in BALF by NK cells following i.n. Adv vaccination

(A–C) NK cell-depleted WT mice by anti-NK1.1 antibody were intranasally administered Ad-LacZ at a dose of 1.0×10^{10} VP/mouse on days 0 and 14. Titers of anti-β-gal (A) IgG and (B) IgA in BALF, and of (C) anti-β-gal IgG in serum were measured on day 21. (D and E) β-Gal expression in the lung was measured at the mRNA and protein levels. (D) At 48 h after i.n. Ad-LacZ vaccination, total RNA was extracted from lungs, and mRNA expression of β-gal was measured by RT-qPCR and normalized against *Gapdh* expression. Graphs represent the relative mRNA expression of each gene normalized against its expression in isotype. (E) β-Gal activity levels in the lung were determined 48 h after administration. Data are pooled (A–E) from two independent experiments and are shown as the mean ± SEM. (A–C) PBS ($n = 3$), Ad ($n = 9$); (D) isotype ($n = 8$), anti-NK1.1 ($n = 8$); and (E) PBS ($n = 3$), Ad ($n = 8$). $^{*}p < 0.05$.

of virus particle and infected cells.[33] Thus, the contributions of alveolar macrophages should be considered in the suppression of Ag-specific antibody production. Moreover, it has been reported that several ISGs produced downstream of type I IFN signaling are involved in suppressing viral protein translation.[34] The impact of ISGs will be further investigated to clarify their role in the immune response mechanisms associated with i.n. Adv vaccination. In terms of a vaccine design, our findings suggest that type I IFN signaling limits vaccine-induced Ag-specific immune responses following i.n. Adv vaccination. Accordingly, the strategies of neutralizing type I IFN, such as the vaccinia virus-derived soluble type I IFN decoy receptor B18R[35,36] or the expression of a compact IFNAR-targeting single-chain fragment variable,[37,38] could enable transient blockade of type I IFN signaling. These would enhance the Ag-specific antibody production to inhibit type I IFN-activated NK cells. Since this study evaluated the vaccine effects against model Ag, β-gal, future studies should evaluate the effects on infection prevention of Adv vaccines using clinically relevant Ags, such as SARS-CoV-2 spike or influenza hemagglutinin.

In summary, this study demonstrated that NK cells activated by type I IFN signaling suppress Ag-specific antibody production after i.n. Adv vaccination. These findings contribute to the elucidation of the innate immune activation and Ag-specific immune response mechanisms following i.n. Adv vaccination and promote the development of more protective and safer Adv vaccines.

MATERIALS AND METHODS

Mice

C57BL/6J WT mice were purchased by Japan SLC (Hamamatsu, Japan), and *Ifnar1*$^{-/-}$ mice (C57BL/6J background) were established as described previously.[39] All mice were bred in an animal facility under specific pathogen-free conditions, and 6- to 8-week-old female mice were used for experiments. All of the animal experiments performed in this study were approved by the Animal Experiment Committee of The University of Osaka (approval no. 28-3-3).

Adv production

β-Gal, encoded by *lacZ*, was used as a model Ag. Ad-LacZ was constructed as described previously.[40,41] Briefly, the expression cassette containing the cytomegalovirus promoter-driven *lacZ* gene was inserted into the E1-deletion region of the E1/E3-deleted adenovirus type 5 genome. This virus was propagated in HEK293 cells, purified by two rounds of cesium chloride-gradient ultracentrifugation, dialyzed, and stored at −80°C. Determination of the virus particle (VP) titers was accomplished spectrophotometrically according to the methods of Maizel et al.[42]

Induction of anti-β-gal immunity in mice and collection of serum and BALF

Ad-LacZ (1×10^{10} VP/30 μL/mouse) was administered intranasally (1×10^{10} VP/30 μL/mouse) or intramuscularly (1×10^{10} VP/50 μL/mouse) into WT and *Ifnar1*$^{-/-}$ mice (female, 6–8 weeks old) on days 0 and 14. On day 21, serum and BALF were collected. Orbital blood was allowed to clot for 1 h at room temperature, followed by centrifugation at 6,200 × *g* for 20 min at 4°C, and the supernatant was collected as serum. BALF was collected by injecting 1.2 mL of PBS into the lungs through the trachea using a cannula (Terumo, Tokyo, Japan, #SR-FF2225). The BALF was the supernatant collected after centrifugation at 6,200 × *g* for 20 min at 4°C. Each sample was stored at −80°C.

β-Gal activity assay

The lungs were isolated from the mice and homogenized in lysis buffer (0.05% Triton X-100, 2 mM EDTA, 0.1 M Tris [pH 7.8]), using

Table 1. The primers used for quantitative RT-PCR

Gene	Forward primer	Reverse primer
Gapdh	CCAGGTTGTCTCCTGCGACTT	CCTGTTGCTGTAGCCGTATTCA
β-gal	AACTATCCCGACCGCCTTACTG	TAGCGGCTGATGTTGAACTG
Gzmb	GAAGATCCTCCTGCTACTGCTG	GCACAAAGTCCTCTCGAATAAGG
Prf1	CTGTTCCTCCTGGGCCTTTT	TGGCACGAACTTGTGCTTCT
Ifng	GGATGGTGACATGAAAATCCTGC	TGCTGATGGCCTGATTGTCTT
Cxcl9	GGAGTTCGAGGAACCCTAGTG	GGGATTTGTAGTGGATCGTGC
Cxcl10	CCAAGTGCTGCCGTCATTTTC	GGCTCGCAGGGATGATTTCAA
Cxcl11	GGCTTCCTTATGTTCAAACAGGG	GCCGTTACTCGGGTAAATTACA

Micro Smash (MS-100; Tomy Digital Biology, Tokyo, Japan) 48 h after administration of Ad-LacZ, followed by three rounds of freezing and thawing. The homogenates were then centrifuged at 21,900 × *g* for 10 min at 4°C. β-Gal activity in the supernatants was measured using a Galacto-Light Plus System (Applied Biosystems, Bedford, MA) with a luminometer (Lumat LB 9507, Berthold, Bad Wildbad, Germany). The protein concentrations in the lung suspension were measured using the Bio-Rad Protein Assay Kit (Bio-Rad, Hercules, CA) on a plate reader (iMark, Bio-Rad) and utilized to correct for β-gal activity.

Enzyme-linked immunosorbent assay

Anti-β-gal antibody titers in the serum and BALF were determined by enzyme-linked immunosorbent assay (ELISA) as follows. The 96-well plates were coated with recombinant β-gal (0.025 mg/mL) (Corning, Corning, NY) in carbonate buffer overnight at 4°C. The coated plates were then incubated with 5-fold diluted ImmunoBlock (DS Pharma Biomedical, Osaka, Japan) for 1 h at room temperature. Samples that were diluted using 20-fold diluted ImmunoBlock were added to the Ag-coated plates and incubated for 2 h at 37°C, followed by 2-h incubation with goat anti-mouse whole IgG BIOT or goat anti-mouse IgA BIOT (dilution 1/5,000; Southern Biotech, Birmingham, AL) at 37°C. After incubation with the secondary antibody, horseradish peroxidase-labeled streptavidin (dilution 1/5,000; Southern Biotech) was added and incubated for 1 h at room temperature. After incubation, the color reaction was developed using a Substrate Reagent Pack (R&D System, Minneapolis, MN, #DY999) and stopped with 0.5 N HCl, and the absorbance was measured at OD_{450} on a plate reader (TriStar LB 941, Berthold).

RNA isolation and quantitative reverse-transcription polymerase chain reaction

After the lungs were isolated and homogenized 24 h after administration of Ad-LacZ, total RNA was extracted using ISOGEN (Nippon Gene, Tokyo, Japan). Complementary DNA (cDNA) was synthesized from total RNA that had been treated with RNase-free DNase I (New England Biolabs, Ipswich, MA), using the SuperScript VILO cDNA Synthesis Kit (Thermo Fisher Scientific, Waltham, MA). Quantitative reverse-transcription polymerase chain reaction (RT-qPCR) was performed using cDNA as a template and the THUNDERBIRD Next SYBR qPCR Mix (TOYOBO, Osaka, Japan) with a StepOnePlus Real-Time PCR System (Applied Biosystems). Relative expression was calculated using the ΔΔCt method, and the mRNA level of each gene was normalized to that of *Gapdh*. The primer sequences used in this study are shown in Table 1.

Isolation of lymphocytes

The lungs were cut into small pieces and digested in 2% FBS/PBS supplemented with DNase I (New England Biolabs) and 100 U/mL collagenase type I (Fujifilm Wako Pure Chemical Industries, Osaka, Japan) at 37°C for 80 min with gentle agitation. The digested lungs were dissociated by extrusion through a 70-μm cell strainer (AS ONE, Osaka, Japan) and washed with 2% FBS/PBS. Red blood cells were lysed, and the precipitates were then suspended in RPMI-1640 (Sigma-Aldrich, St. Louis, MO, USA). The cell suspension was layered over Lympholyte-M (Cedarlane, Burlington, ON, Canada) and centrifuged at 1,200 × *g* for 20 min at room temperature. Cells at the interface between RPMI-1640 and Lympholyte-M were collected as viable lung lymphocytes.

Flow cytometry

Isolated cells were treated with mouse FcR blocking reagent (Miltenyi Biotec, Bergisch Gladbach, Germany) at 4°C for 5 min. Fluorescently labeled antibodies shown in Table 2 were then added and stained at 4°C for 30 min. Dead cells were excluded by staining with 7-amino-actinomycin D (Thermo Fisher Scientific). The stained samples were acquired using a MACSQuant flow cytometer (Miltenyi Biotec), and samples were analyzed with FlowJo software v.10 (FlowJo, LLC, Ashland, OR). Gating was performed as shown in Figure S4A.

NK cell depletion

NK cells were depleted using 100 μg/mouse of anti-NK1.1 (clone PK136; Selleck, Houston, TX) in C57BL/6J mice. Mice were administered intranasally with 1 × 10^{10} VP Ad-LacZ on days 0 and 14 and treated with intraperitoneal injections of isotype control IgG2a (clone C1.18.4; Selleck) or anti-NK1.1 on day −1 and every 3 days thereafter until the termination of the experiment. NK cell depletion in the lungs was confirmed by flow cytometry (Figures S4B and S4C).

Table 2. List of antibodies used for flow cytometry

Target	Species	Clone	Fluorochrome	Manufacturer
CD3ε	Hamster anti-mouse	145-2C11	FITC	BioLegend
CD45	Rat anti-mouse	S18009F	Pacific Blue	BioLegend
NK1.1	Mouse anti-mouse	PK136[a] S17016D[a]	APC	BioLegend
NKG2D	Rat anti-mouse	CX5	PE	BioLegend

APC, allophycocyanin; FITC, fluorescein isothiocyanate; PE, phycoerythrin.
[a]PK136 was used for NK cell analysis, and S17016D was used to confirm NK cell depletion.

Statistical analyses

Two-way ANOVA followed by Tukey's post hoc test was used for statistical analyses. Also, Mann-Whitney U test was only used to compare β-gal activity. These analyses were conducted using GraphPad Prism software (GraphPad Software, San Diego, CA). Data are presented as means ± SEM.

DATA AND CODE AVAILABILITY

All data generated or analyzed during this study are included in this article and supplemental information.

ACKNOWLEDGMENTS

We thank Drs. Eiko Sakai and Yukiko Toba-Ueyama (Graduate School of Pharmaceutical Sciences, The University of Osaka, Osaka, Japan), Toshiro Hirai, and Yasuo Yoshioka (Institute for Open and Transdisciplinary Research Initiatives, The University of Osaka, Osaka Japan) for their support. This study was supported by grants-in-aid for Scientific Research (A) (23H00552) from the Ministry of Education, Culture, Sports, Science and Technology (MEXT) of Japan and the Platform Project for Supporting Drug Discovery and Life Science Research (Basis for Supporting Innovative Drug Discovery and Life Science Research [BINDS]) from AMED (grant numbers JP25ama121052 and JP25ama121054). This study was supported by JST SPRING, grant number JPMJSP2138. This study was partially supported by BIKEN Foundation. H.N. received BIKEN Taniguchi Scholarship from BIKEN Foundation. R.O. is Research Fellow of the Japan Society for the Promotion of Science.

AUTHOR CONTRIBUTIONS

H.N., data curation, formal analysis, investigation, methodology, validation, visualization, writing – original draft, and writing – review & editing; M.T., methodology, supervision, and writing – review & editing; R.O., investigation and methodology. T.N., investigation; K.J.I., resources; K.S., supervision; F.S., supervision; H.M., conceptualization, funding acquisition, supervision, and writing – review & editing.

DECLARATION OF INTERESTS

The authors declare no competing interests.

SUPPLEMENTAL INFORMATION

Supplemental information can be found online at https://doi.org/10.1016/j.omta.2026.201742.

REFERENCES

1. Gallo, O., Locatello, L.G., Mazzoni, A., Novelli, L., and Annunziato, F. (2021). The central role of the nasal microenvironment in the transmission, modulation, and clinical progression of SARS-CoV-2 infection. Mucosal Immunol. *14*, 305–316. https://doi.org/10.1038/S41385-020-00359-2.
2. Neutra, M.R., and Kozlowski, P.A. (2006). Mucosal vaccines: the promise and the challenge. Nat. Rev. Immunol. *6*, 148–158. https://doi.org/10.1038/nri1777.
3. Xing, M., Hu, G., Wang, X., Wang, Y., He, F., Dai, W., Wang, X., Niu, Y., Liu, J., Liu, H., et al. (2024). An intranasal combination vaccine induces systemic and mucosal immunity against COVID-19 and influenza. NPJ Vaccines *9*, 64. https://doi.org/10.1038/S41541-024-00857-5.
4. Rudenko, L., Desheva, J., Korovkin, S., Mironov, A., Rekstin, A., Grigorieva, E., Donina, S., Gambaryan, A., and Katlinsky, A. (2008). Current status of live attenuated influenza vaccine in the United States for seasonal and pandemic influenza. Influenza Other Respir. Viruses *2*, 193–202. https://doi.org/10.1111/j.1750-2659.2008.00056.x.
5. Halperin, S.A., Ye, L., MacKinnon-Cameron, D., Smith, B., Cahn, P.E., Ruiz-Palacios, G.M., Ikram, A., Lanas, F., Lourdes Guerrero, M., Muñoz Navarro, S.R., et al.; CanSino COVID-19 Global Efficacy Study Group (2022). Final efficacy analysis, interim safety analysis, and immunogenicity of a single dose of recombinant novel coronavirus vaccine (adenovirus type 5 vector) in adults 18 years and older: an international, multicentre, randomised, double-blinded, placebo-con. Lancet *399*, 237–248. https://doi.org/10.1016/S0140-6736(21)02753-7.
6. Akula, V.R., Bhate, A.S., Gillurkar, C.S., Kushwaha, J.S., Singh, A.P., Singh, C., Pandey, A.K., K. K, S., Rai, S.K., and Vadrevu, K.M.; BBV154 Study Group (2025). Effect of heterologous intranasal iNCOVACC® vaccination as a booster to two-dose intramuscular Covid-19 vaccination series: a randomized phase 3 clinical trial. Commun. Med. *5*, 133. https://doi.org/10.1038/s43856-025-00818-6.
7. Kawai, T., and Akira, S. (2011). Toll-like Receptors and Their Crosstalk with Other Innate Receptors in Infection and Immunity. Immunity *34*, 637–650. https://doi.org/10.1016/J.IMMUNI.2011.05.006.
8. Pulendran, B., and Ahmed, R. (2006). Translating Innate Immunity into Immunological Memory: Implications for Vaccine Development. Cell *124*, 849–863. https://doi.org/10.1016/J.CELL.2006.02.019.
9. Pulendran, B., and Ahmed, R. (2011). Immunological mechanisms of vaccination. Nat. Immunol. *12*, 509–517. https://doi.org/10.1038/ni.2039.
10. Hendrickx, R., Stichling, N., Koelen, J., Kuryk, L., Lipiec, A., and Greber, U.F. (2014). Innate Immunity to Adenovirus. Hum. Gene Ther. *25*, 265–284. https://doi.org/10.1089/HUM.2014.001.
11. Nociari, M., Ocheretina, O., Schoggins, J.W., and Falck-Pedersen, E. (2007). Sensing Infection by Adenovirus: Toll-Like Receptor-Independent Viral DNA Recognition Signals Activation of the Interferon Regulatory Factor 3 Master Regulator. J. Virol. *81*, 4145–4157. https://doi.org/10.1128/jvi.02685-06.
12. Lam, E., Stein, S., and Falck-Pedersen, E. (2014). Adenovirus Detection by the cGAS/STING/TBK1 DNA Sensing Cascade. J. Virol. *88*, 974–981. https://doi.org/10.1128/jvi.02702-13.
13. Tatsis, N., and Ertl, H.C.J. (2004). Adenoviruses as vaccine vectors. Mol. Ther. *10*, 616–629. https://doi.org/10.1016/J.YMTHE.2004.07.013.
14. Medzhitov, R., and Janeway, C. (2000). Innate immune recognition: mechanisms and pathways. Immunol. Rev. *173*, 89–97. https://doi.org/10.1034/J.1600-065X.2000.917309.X.
15. Yamaguchi, T., Kawabata, K., Kouyama, E., Ishii, K.J., Katayama, K., Suzuki, T., Kurachi, S., Sakurai, F., Akira, S., and Mizuguchi, H. (2010). Induction of type I interferon by adenovirus-encoded small RNAs. Proc. Natl. Acad. Sci. USA *107*, 17286–17291. https://doi.org/10.1073/PNAS.1009823107.
16. Hensley, S.E., Giles-Davis, W., McCoy, K.C., Weninger, W., and Ertl, H.C.J. (2005). Dendritic Cell Maturation, but Not CD8+ T Cell Induction, Is Dependent on Type I IFN Signaling during Vaccination with Adenovirus Vectors. J. Immunol. *175*, 6032–6041. https://doi.org/10.4049/JIMMUNOL.175.9.6032.
17. Mueller, S.N., and Germain, R.N. (2009). Stromal cell contributions to the homeostasis and functionality of the immune system. Nat. Rev. Immunol. *9*, 618–629. https://doi.org/10.1038/nri2588.
18. Zhu, J., Huang, X., and Yang, Y. (2008). A Critical Role for Type I IFN–dependent NK Cell Activation in Innate Immune Elimination of Adenoviral Vectors In Vivo. Mol. Ther. *16*, 1300–1307. https://doi.org/10.1038/MT.2008.88.
19. Lee, A.J., and Ashkar, A.A. (2018). The dual nature of type I and type II interferons. Front. Immunol. *9*, 2061. https://doi.org/10.3389/FIMMU.2018.02061/EPUB.
20. Quinn, K.M., Zak, D.E., Costa, A., Yamamoto, A., Kastenmuller, K., Hill, B.J., Lynn, G.M., Darrah, P.A., Lindsay, R.W.B., Wang, L., et al. (2015). Antigen expression

determines adenoviral vaccine potency independent of IFN and STING signaling. J. Clin. Investig. *125*, 1129–1146. https://doi.org/10.1172/JCI78280.

21. Johnson, M.J., Petrovas, C., Yamamoto, T., Lindsay, R.W.B., Loré, K., Gall, J.G.D., Gostick, E., Lefebvre, F., Cameron, M.J., Price, D.A., et al. (2012). Type I IFN Induced by Adenovirus Serotypes 28 and 35 Has Multiple Effects on T Cell Immunogenicity. J. Immunol. *188*, 6109–6118. https://doi.org/10.4049/jimmunol.1103717.

22. Carlin, L.E., Hemann, E.A., Zacharias, Z.R., Heusel, J.W., and Legge, K.L. (2018). Natural killer cell recruitment to the lung during influenza A virus infection is dependent on CXCR3, CCR5, and virus exposure dose. Front. Immunol. *9*, 781. https://doi.org/10.3389/FIMMU.2018.00781/BIBTEX.

23. Lee, S.H., Miyagi, T., and Biron, C.A. (2007). Keeping NK cells in highly regulated antiviral warfare. Trends Immunol. *28*, 252–259. https://doi.org/10.1016/J.IT.2007.04.001.

24. Hashimoto, S., Hirai, T., Ueda, K., Kakihara, M., Tokunoh, N., Ono, C., Matsuura, Y., Takayama, K., and Yoshioka, Y. (2025). Hypertonic intranasal vaccines gain nasal epithelia access to exert strong immunogenicity. Mucosal Immunol. *18*, 793–809. https://doi.org/10.1016/j.mucimm.2025.03.006.

25. Ko, K.H., Bae, H.S., Lee, S.M., Park, S., Han, S.H., Heo, J., Kim, J., Cho, Y., Kim, D.-H., and Cha, S.B. (2025). Intranasal vaccines adjuvanted with Nexavant demonstrate robust protective efficacy by inducing both mucosal and systemic immunity in a murine model. Front. Immunol. *16*, 1745319. https://doi.org/10.3389/fimmu.2025.1745319.

26. Li, J., Xiao, C., Li, C., and He, J. (2025). Tissue-resident immune cells: from defining characteristics to roles in diseases. Signal Transduct. Targeted Ther. *10*, 12. https://doi.org/10.1038/s41392-024-02050-5.

27. Chen, Z., Quan, L., Huang, A., Zhao, Q., Yuan, Y., Yuan, X., Shen, Q., Shang, J., Ben, Y., Qin, F.X.F., and Wu, A. (2018). seq-ImmuCC: Cell-centric view of tissue transcriptome measuring cellular compositions of immune microenvironment from mouse RNA-seq data. Front. Immunol. *9*, 1286. https://doi.org/10.3389/fimmu.2018.01286.

28. Yu, Y.R.A., O'Koren, E.G., Hotten, D.F., Kan, M.J., Kopin, D., Nelson, E.R., Que, L., and Gunn, M.D. (2016). A Protocol for the Comprehensive Flow Cytometric Analysis of Immune Cells in Normal and Inflamed Murine Non-Lymphoid Tissues. PLoS One *11*, e0150606. https://doi.org/10.1371/journal.pone.0150606.

29. Moyron-Quiroz, J.E., Rangel-Moreno, J., Kusser, K., Hartson, L., Sprague, F., Goodrich, S., Woodland, D.L., Lund, F.E., and Randall, T.D. (2004). Role of inducible bronchus associated lymphoid tissue (iBALT) in respiratory immunity. Nat. Med. *10*, 927–934. https://doi.org/10.1038/nm1091.

30. Moyron-Quiroz, J.E., Rangel-Moreno, J., Hartson, L., Kusser, K., Tighe, M.P., Klonowski, K.D., Lefrançois, L., Cauley, L.S., Harmsen, A.G., Lund, F.E., and Randall, T.D. (2006). Persistence and Responsiveness of Immunologic Memory in the Absence of Secondary Lymphoid Organs. Immunity *25*, 643–654. https://doi.org/10.1016/j.immuni.2006.08.022.

31. Fehniger, T.A., Cai, S.F., Cao, X., Bredemeyer, A.J., Presti, R.M., French, A.R., and Ley, T.J. (2007). Acquisition of Murine NK Cell Cytotoxicity Requires the Translation of a Pre-existing Pool of Granzyme B and Perforin mRNAs. Immunity *26*, 798–811. https://doi.org/10.1016/J.IMMUNI.2007.04.010.

32. Wang, J., Li, F., Zheng, M., Sun, R., Wei, H., and Tian, Z. (2012). Lung natural killer cells in mice: phenotype and response to respiratory infection. Immunology *137*, 37–47. https://doi.org/10.1111/J.1365-2567.2012.03607.X.

33. Worgall, S., Leopold, P.L., Wolff, G., Ferris, B., Van Roijen, N., and Crystal, R.G. (1997). Role of alveolar macrophages in rapid elimination of adenovirus vectors administered to the epithelial surface of the respiratory tract. Hum. Gene Ther. *8*, 1675–1684. https://doi.org/10.1089/hum.1997.8.14-1675.

34. Ivashkiv, L.B., and Donlin, L.T. (2014). Regulation of type I interferon responses. Nat. Rev. Immunol. *14*, 36–49. https://doi.org/10.1038/nri3581.

35. Symons, J.A., Alcamí, A., and Smith, G.L. (1995). Vaccinia virus encodes a soluble type I interferon receptor of novel structure and broad species soecificity. Cell *81*, 551–560. https://doi.org/10.1016/0092-8674(95)90076-4.

36. Colamonici, O.R., Domanski, P., Sweitzer, S.M., Larner, A., and Buller, R.M. (1995). Vaccinia Virus B18R Gene Encodes a Type I Interferon-binding Protein That Blocks Interferon α Transmembrane Signaling. J. Biol. Chem. *270*, 15974–15978. https://doi.org/10.1074/jbc.270.27.15974.

37. Huston, J.S., Levinson, D., Mudgett-Hunter, M., Tai, M.S., Novotný, J., Margolies, M.N., Ridge, R.J., Bruccoleri, R.E., Haber, E., Crea, R., et al. (1988). Protein engineering of antibody binding sites: Recovery of specific activity in an anti-digoxin single-chain Fv analogue produced in Escherichia coli. Proc. Natl. Acad. Sci. USA *85*, 5879–5883. https://doi.org/10.1073/pnas.85.16.5879.

38. Wang, F., Zhang, Q., Zhang, F., Zhang, E., Li, M., Ma, S., Guo, J., Yang, Z., and Zhu, J. (2023). Adenovirus vector-mediated single chain variable fragments target the nucleocapsid protein of porcine epidemic diarrhea virus and protect against viral infection in piglets. Front. Immunol. *14*, 1058327. https://doi.org/10.3389/fimmu.2023.1058327.

39. Ishii, K.J., Coban, C., Kato, H., Takahashi, K., Torii, Y., Takeshita, F., Ludwig, H., Sutter, G., Suzuki, K., Hemmi, H., et al. (2006). A toll-like receptor-independent antiviral response induced by double-stranded B-form DNA. Nat. Immunol. *7*, 40–48. https://doi.org/10.1038/NI1282.

40. Kawabata, K., Sakurai, F., Yamaguchi, T., Hayakawa, T., and Mizuguchi, H. (2005). Efficient gene transfer into mouse embryonic stem cells with adenovirus vectors. Mol. Ther. *12*, 547–554. https://doi.org/10.1016/j.ymthe.2005.04.015.

41. Hemmi, M., Tachibana, M., Tsuzuki, S., Shoji, M., Sakurai, F., Kawabata, K., Kobiyama, K., Ishii, K.J., Akira, S., and Mizuguchi, H. (2014). The Early Activation of CD8+ T Cells Is Dependent on Type I IFN Signaling following Intramuscular Vaccination of Adenovirus Vector. Biomed Res. Int. *2014*, 158128. https://doi.org/10.1155/2014/158128.

42. Maizel, J.V., White, D.O., and Scharff, M.D. (1968). The polypeptides of adenovirus: I. Evidence for multiple protein components in the virion and a comparison of types 2, 7A, and 12. Virology *36*, 115–125. https://doi.org/10.1016/0042-6822(68)90121-9.

OMTA, Volume *34*

Supplemental information

Efficacy of intranasal adenovirus vector vaccine is attenuated by type I IFN-induced NK cell activation

Hayato Nakatani, Masashi Tachibana, Rika Onishi, Takato Nakagaki, Ken J. Ishii, Kahori Shimizu, Fuminori Sakurai, and Hiroyuki Mizuguchi

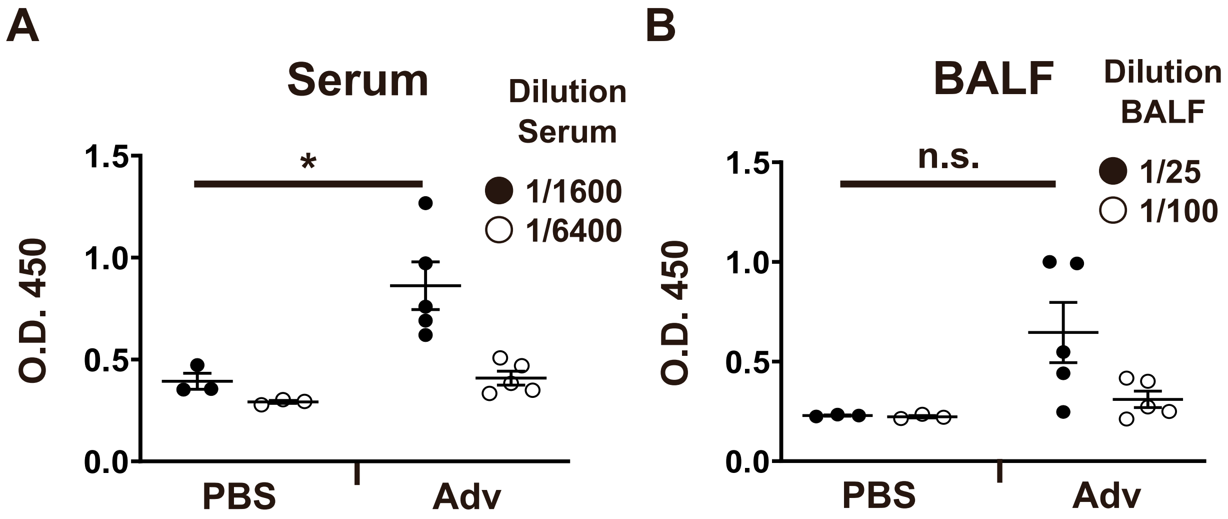

Figure S1. The induction of anti-Ad IgG in serum and BALF following *i.n.* Adv vaccination. (A, B) WT mice were administered Ad-LacZ *i.n*. at a dose of 1.0 × 10^{10} VP/mouse on days 0 and 14. Titers of anti-Ad IgG in (A) serum and (B) BALF on day 21 were measured by ELISA using 96-well plates coated with a replication-incompetent Ad5 vector encoding no transgene (5 × 10^6 VP/well). Data are pooled (A, B) from one independent experiment and are shown as the mean ± S.E.M. (A, B) PBS (n = 3), Adv (n = 5). *$p < 0.05$.

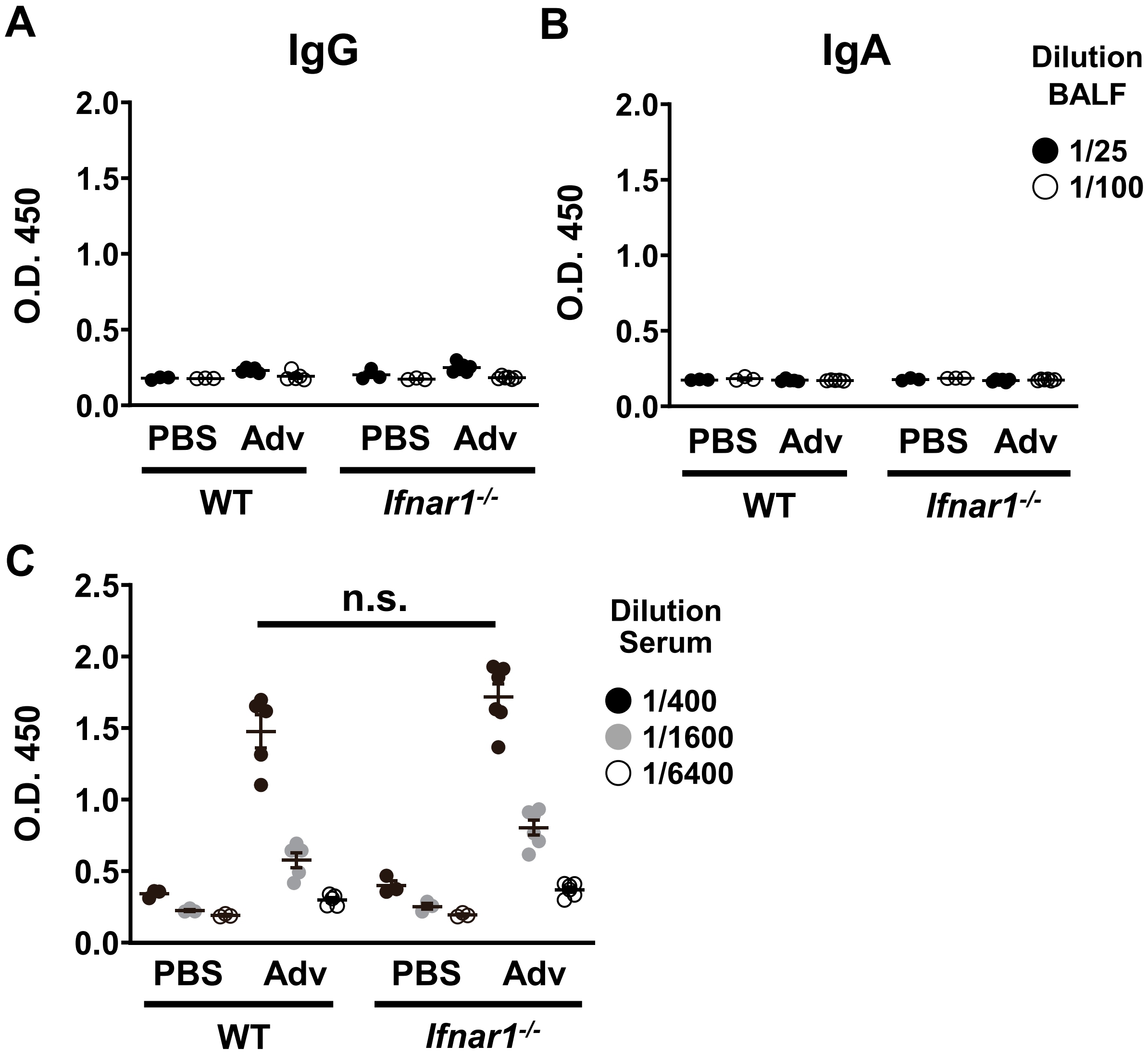

Figure S2. Type I IFN signaling does not contribute to β-gal–specific antibody production at respiratory mucosal surfaces following *i.m.* Adv vaccination. (A-C) WT and *Ifnar1$^{-/-}$* mice were administered Ad-LacZ *i.m.* at a dose of 1.0×10^{10} VP/mouse on days 0 and 14. Titers of anti-β-gal (A) IgG and (B) IgA in BALF and of (C) anti-β-gal IgG in serum were measured on day 21. Data are pooled (A-C) from two independent experiments and are shown as the mean ± S.E.M. (A-C) PBS (n = 3), WT + Ad (n = 5), *Ifnar1$^{-/-}$* + Ad (n = 6).

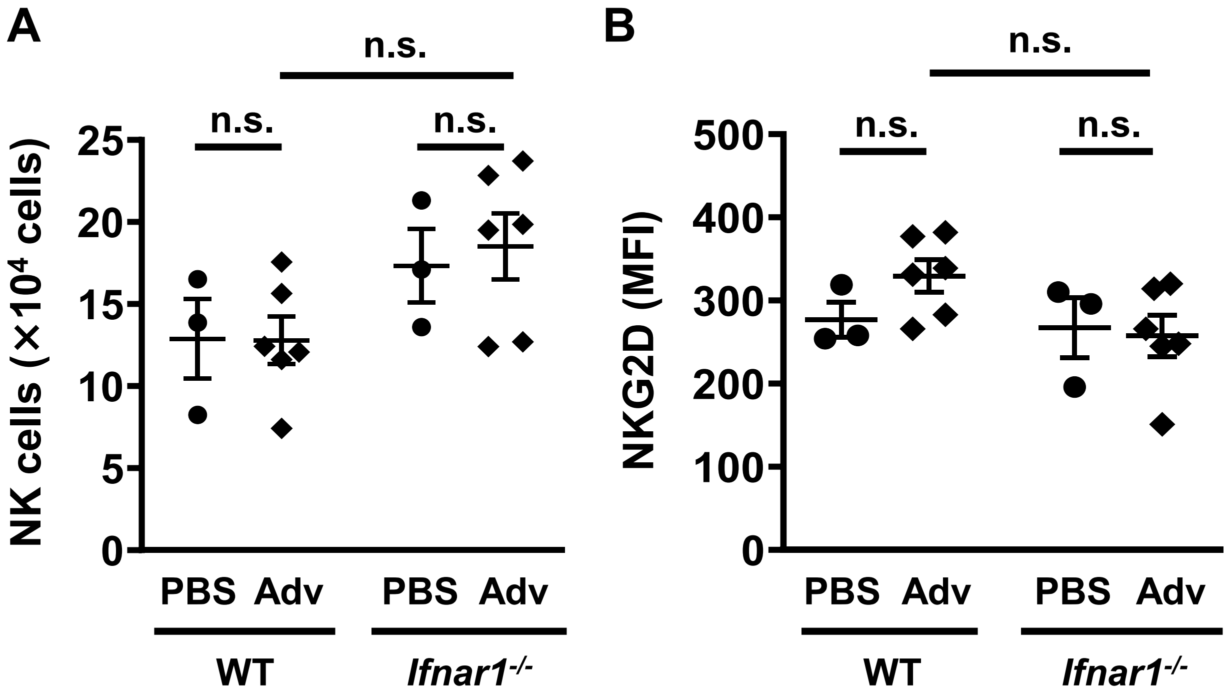

Figure S3. NK cells were not activated in the spleen after *i.n.* Adv vaccination. (A, B) WT and *Ifnar1*$^{-/-}$ mice were *i.n.* administered Ad-LacZ at a dose of 1.0 × 10^{10} VP/mouse on day 0. In the spleen after 3 days of *i.n.* administration, (A) NK cell (NK1.1^{+}CD3^{-}) number, and (B) the expression of NKG2D, assessed by mean fluorescence intensity (MFI) as a marker of NK cell activation, were measured by flow cytometry. Data are pooled (A, B) from two independent experiments and are shown as the mean ± S.E.M. (A, B) PBS (n = 3), Ad (n = 6). *$p < 0.05$.

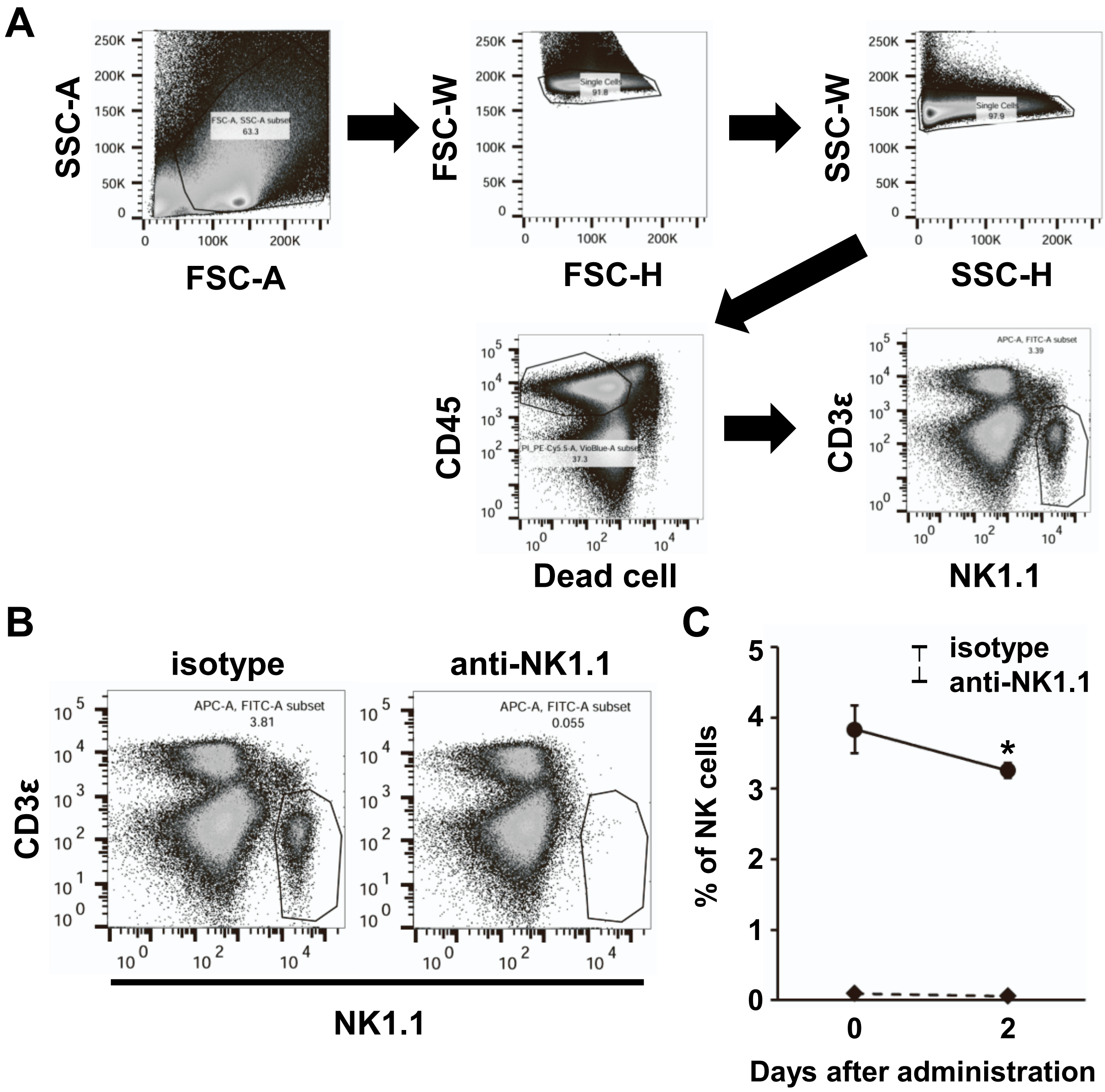

Figure S4. Flow cytometry gating strategy and NK cell depletion in the lung. (A) The flow cytometry gating strategy is illustrated. (B) NK cell populations in the lungs of WT mice treated with either IgG2a isotype control or anti-NK1.1 were compared. (C) WT mice were treated with isotype control or anti-NK1.1 on day -1. Lungs were excised on days 0 and 2, and the percentage of NK cells among $CD45^+$ cells was measured by flow cytometry. Data are pooled (C) from two independent experiments and are shown as the mean ± S.E.M. (C) isotype (n = 3-4), anti-NK1.1 (n = 4). *$p < 0.05$.
